# Exploring the educational needs of transgender and non-binary healthcare among Polish medical students

**DOI:** 10.1186/s12909-026-10139-4

**Published:** 2026-08-14

**Authors:** Patrycja Bździuch, Claudia Sieńko, Jakub Buziak, Joanna Brodowska, Anna Torres

**Affiliations:** 1 Student Scientific Association, Department of Paediatric and Adolescent Gynaecology, Medical University of Lublin, Profesora Antoniego Gębali 6, Lublin, 20-093 Poland; 2 Department of Paediatric and Adolescent Gynaecology, Children’s University Hospital, Profesora Antoniego Gębali 6, Lublin, 20-093 Poland

**Keywords:** Educational needs, Medical education, Transgender, Gender diverse, Transgender healthcare, Survey, Medical students

## Abstract

**Background:**

Given that the transgender and non-binary community now accounts for as much as 1.2% of the total population, they are becoming an increasingly visible subgroup of patients in the healthcare system. At the same time, research indicates that these patients often experience poorer treatment outcomes or have limited access to services. Discrepancies between medical education and subsequent patient care are addressed by incorporating comprehensive education into medical training. To the best of our knowledge, this is one of the first studies exploring healthcare students’ needs regarding transgender healthcare content in medical education in Poland. The aim of the study is to investigate healthcare students’ knowledge of and attitudes towards transgender and non-binary (TGNB) patients, their preparedness to provide healthcare for this population, and their perceived educational needs in this area.

**Methods:**

An online survey on educational needs in healthcare of TGNB people was disseminated through social media and posters with a QR code. The survey was anonymous and targeted healthcare students from Poland. For open-ended questions, an inductive thematic analysis was performed.

**Results:**

Two hundred and sixty-four students, from medical-related faculties, completed the questionnaire. Two hundred and thirty-two (87.9%) respondents admitted that they had not received any content on healthcare for TGNB patients during their studies to date. One hundred and sixty-one (61.0%) of the respondents believed that content on TGNB patient care should be included in the curriculum. Communication with patients (73.9%) was indicated as the most relevant issues in the care of TGNB patients. Respondents rated their knowledge and skills as lowest in surgical treatment (59.1%) and hormonal treatment (50.0%). One hundred and twenty students (45.5%) did not feel prepared to interact with TGNB patients.

**Conclusions:**

Polish healthcare students express an interest in improving their knowledge and skills on the healthcare of TGNB patients. Implementing educational interventions is essential to reduce future gap in medical services access for these patients.

**Trial registration:**

The decision number of the Bioethical Committee of the Medical University of Lublin is KE/138/02/24, Date of determination February 29, 2024.

**Supplementary Information:**

The online version contains supplementary material available at https://doi.org/10.1186/s12909-026-10139-4.

## Background

The term ‘transgender and non-binary’ (TGNB) refers to all individuals whose gender identity and/or expression is different from that assigned at birth [1]. TGNB people include transgender individuals whose gender identities and/or behaviours differ from those culturally associated with their assigned sex, as well as non-binary and genderqueer (NBGQ) people who do not identify within the gender binary system (male vs. female) [2]. Studies from many countries around the world have estimated the prevalence of transgender and gender-diverse people to be between 0.5% and 1.2% of the general population [3, 4]. Importantly, the World Health Organization relocated gender incongruence from the chapter on Mental and Behavioral Disorders to the new section on Conditions Related to Sexual Health in the 11th revision of the International Classification of Diseases, reflecting a shift away from pathologizing of gender diversity [5]. Simultaneously, research has consistently shown that transgender and gender-diverse individuals experience distinct health risks and worse health outcomes compared to their cisgender peers [6, 7]. Documented health disparities include higher rates of certain cancers, poorer cardiovascular health, and an increased prevalence of chronic illnesses, sexually transmitted infections, substance use disorders, and mental health conditions [6–8]. Evidence also suggests that non-binary individuals may experience unique or even greater health disparities compared to binary transgender individuals [9]. One of the contributing factors to these disparities is the experience of discrimination within healthcare systems. The 2022 U.S. Transgender Survey found that only 8.0% of participants reported that their primary care physician knew “almost everything” or “most things” about transgender healthcare [10]. Studies have described less comfort and knowledge related to caring for transgender individuals compared to caring for lesbian, gay, and bisexual (LGB) patients [11, 12]. Clinicians who lack adequate education regarding gender diversity or who hold explicit or implicit biases may engage in discriminatory behaviors or use inappropriate language [13]. Such interactions can reinforce institutional and systemic discrimination, sometimes leading to the denial or invalidation of transgender and gender-diverse identities [13, 14]. As a consequence, affected individuals may delay or avoid seeking healthcare, resulting in poorer health outcomes and reduced access to services comparable to those available to the general population [7, 13]. For these reasons, the implementation of comprehensive and evidence-based education on transgender and gender-diverse health issues during undergraduate and postgraduate medical training is essential to reduce health disparities and improve the quality of care provided to this population [5, 7, 13]. Data regarding the presence of TGNB-related content in Polish medical curricula are limited, in part because each medical university in Poland independently determines the syllabi for clinical subjects. Analysis of the 2021–2027 medical curriculum at the Medical University of Lublin revealed that TGNB topics appeared in only one subject — Psychosocial Aspects of Medicine, where a single 3-hour session addressed sexual dysfunctions and psychomedical aspects of gender identity. No TGNB-related content was identified in the Gynaecology and Obstetrics or Endocrinology syllabi (unpublished observations, Medical University of Lublin, 2024). Moreover, other studies revealed that practising Polish endocrinologists did not receive training on transgender patient care during their residency [15]. The growing demand for this type of education has led to an increased need for research on the subject. However, the number of existing studies is still limited. Pioneering research conducted at Stanford University School of Medicine revealed that, across 176 medical schools, most students receive an average of five hours of LGBT-related education followed by rating their curriculum as “fair”, “poor”, or “very poor” [16]. In Poland, the standardization of transgender healthcare started in 2020, when the Polish Sexological Society published the first official guidelines on healthcare for transgender adults [17]. However, no needs assessment studies are available regarding the existing and required educational opportunities on this subject.

In this study, we aimed to examine the attitudes and self-perceived knowledge of students of various healthcare professions. The ultimate purpose of the study is to identify the educational needs of Polish healthcare professions students regarding the healthcare of TGNB patients.

## Methods

The study was conducted using an anonymous questionnaire to ensure respondent privacy and data confidentiality. Data collection was carried out via an electronic survey, which consisted of a total of 21 questions. Based on the literature review regarding the topic, we developed a dedicated survey questionnaire for this study, tailoring the questions to capture the most critical topics in TGNB education at that time. The survey was developed specifically for this study because, to our knowledge, no validated Polish tool addressing medical students’ knowledge, attitudes, and educational needs regarding TGNB health was available at the time. No formal psychometric validation of the questionnaire was performed. However, the questionnaire was reviewed by the authors for clarity, relevance, and consistency with the study objectives. Participation in the survey was restricted to students enrolled in programmes offered at Polish medical universities. The questionnaire was distributed exclusively through closed communication channels in social media controlled by administrators in student year groups and university scientific clubs. Membership in these channels is limited to verified students, and access is granted by designated administrators, such as year representatives and club chairmans. The inclusion criterion was student status in one of the following fields of study typically represented at medical universities: laboratory diagnostics, biomedicine, dietetics, electroradiology, pharmacy, physiotherapy, cosmetology, medicine, dentistry, nursing, midwifery, psychology, paramedics, dental technology, occupational therapy, public health, or healthcare management. To ensure that only eligible respondents completed the survey, a screening question regarding field of study was placed at the very beginning of the questionnaire. The survey was intentionally distributed to all medical professions represented at the medical university, rather than limited to clinical care faculties. This decision was based on the assumption that the act of enrolling in a medical university may be associated with more exposure to medical content in a shared medical academic environment. Participants who indicated enrolment in a programme outside the above scope were excluded from the analysis. Responses were collected anonymously, and completion was confirmed by student representatives at each participating institution. The template of the survey questionnaire is available as Additional file 1. These included both closed-ended (19 questions) and open-ended (2 questions). Six of the closed-ended questions permitted multiple responses. The study includes a qualitative component to provide a better understanding of the respondents’ views on TGNB healthcare education. Using open-ended questions, respondents were invited to justify their answers and explain their reasons for supporting or opposing inclusion. Next, an inductive thematic analysis approach was selected to enable the identification of themes arising directly from participants’ responses without imposing predefined categories [18]. A single answer could be included in several categories at once. The analysis was conducted by two authors (P.B and C.S). Initial analysis of the data performed by each researcher yielded a coding framework of themes, which was used to code the answers in detail during a second round of analysis. In the data analysis process, the coding framework was continuously adapted and discussed with a second researcher. Following the qualitative analysis, a quantitative analysis of the summarized responses was also performed. The study was reviewed by the Bioethics Committee at the Medical University of Lublin (# KE/138/02/24). The survey participants consisted of students from various healthcare professions attending Polish universities. The survey was distributed through on-line student groups in social media.

## Results

### Characteristics of surveyed population

A total of 265 participants completed the survey. One questionnaire was excluded from the analysis because the participant’s study profile did not meet the inclusion criteria. Therefore, 264 surveys were included in the analysis coming from healthcare students of various educational background: medicine (112), physiotherapy (33), midwifery (29), dentistry (21), pharmacy (16), psychology (14), nursing (14), paramedics(12), dietetics (6), electroradiology (2), speech therapy with audiology (1), public health (1), biomedicine (1), laboratory diagnostics (1) and occupational therapy (1). The answers were obtained from students in year I- 19.3% of all responses, year II- 18.9%, year III- 16.7%, year IV- 17.4%, year V- 18.2% and year VI- 9.5%. Among the surveyed academic programs, medicine was the only program with a six-year duration. Using a single self-identified gender question, 250 (94.7%) of the respondents identified themselves as cisgender. Nine (3.4%) respondents identified as non-binary and four (1.5%) as transgender. Participants’ characteristics were presented in Table 1.

**Table 1:** Survey respondents by health professions program, year of study and gender identity (*n* = 264)

		Year of study						Gender identity			
	Number of Survey Respondents	I	II	III	IV	V	VI	Cisgender	Non-binary	Transgender	“I don’t know”
All	264	51	50	44	46	48	25	250	9	4	1
Medicine	112	13	15	13	16	32	23	102	6	1	1
Physiotherapy	33	10	6	2	14	1	0	32	1	0	0
Midwifery	29	10	8	9	1	1	0	29	0	0	0
Dentistry	21	3	3	6	9	0	0	21	0	0	0
Pharmacy	16	1	4	1	2	6	1	15	1	0	0
Psychology	14	1	6	3	0	4	0	13	1	0	0
Nursing	14	5	4	3	1	1	0	13	0	1	0
Paramedics	12	6	2	4	0	0	0	12	0	0	0
Dietetics	6	0	0	2	2	2	0	5	0	1	0
Other Healthcare professions*	7	2	2	1	1	1	0	7	0	**1**	0

*Other Healthcare professions: Electroradiology (*n* = 2), Speech therapy with audiology (*n* = 1), Public health (*n* = 1), Biomedicine (*n* = 1), Laboratory diagnostics (*n* = 1), Occupational therapy (*n* = 1)

Terms “Other Healthcare professions” included Electroradiology, Speech therapy with audiology, Public health, Biomedicine, Laboratory diagnostics and Occupational therapy.

### Opinions about the presence of educational content of healthcare for transgender and non-binary (TGNB) people

Most students (*n* = 161, 61.0%) agreed that TGNB healthcare education should be included in the curricula. Medical students were those who supported this idea the most (68.8%), while the lowest rate of support was found among psychology students (35.8%). Agreement with including TGNB healthcare content in the curriculum was also found to vary depending on the year of study, ranging from 43.5% among fourth-year students to 70.8% among fifth-year students. A detailed breakdown of the responses to the question, categorized by health professions program and year of study, is presented in Tables 2 and 3, respectively. The distribution of responses differed across the six years of the study (χ² = 23.75, df = 10, *p* = 0.008). Fourth-year students showed a different response pattern compared to the other cohorts, reporting the lowest percentage of positive responses (43.5%) and the highest percentage of negative responses (37.0%). After applying the Bonferroni correction for multiple comparisons, only the difference between fourth- and fifth-year students remained statistically significant. The full results of the pairwise comparison are presented in Additional File 2. Those participants, who supported inclusion of TGNB content, further expanded on this point in more detail in open-ended responses. As a result, four main topics were identified: the possibility of an increase of the TGNB healthcare relevance; the need to improve healthcare providers’ skills; improving patient-healthcare professional communication and creating a respectful healthcare environment and improving patient medical treatment outcomes. Students believe that education is needed to provide better communication, respect and comfort for every TGNB patient (*n* = 71). Respondents anticipated the possibility of an increase in the number of patients expressing various gender identities over coming years (*n* = 34). A number of respondents highlighted a widespread lack of knowledge and preparedness among healthcare professionals (*n* = 22). Moreover, participants believed that TGNB healthcare education would have a positive impact on medical treatment outcomes (*n* = 15). The remaining open-ended responses were too heterogeneous to be classified into any of the identified statements (*n* = 19). Unclassified responses were either blank or contained imprecise, general terms such as “awareness,” “the psychology of this population,” or “an important topic”. Sixty three (23.9%) had no opinion. Forty respondents (15.1%) declared that TGNB healthcare education should not be included in the curriculum. They justified this by responding to open-ended questions, which were categorised into four themes: the existence of only two biological sexes; other important topics in the curriculum; the unnecessary nature of additional skills; and the small size of the population. Some of the respondents expressed the opinion that only two genders were biologically justified (*n* = 17). Others justified their choice by the fact that the care and communication with TGNB individuals did not require additional skills from healthcare providers (*n* = 13) and medical curricula should focus on more relevant matters (*n* = 5). The remaining open-ended responses were too heterogeneous to be classified into any of the identified statements (*n* = 3). Some respondents did not justify their choice (*n* = 2).

**Table 2:** Answers to the question: ‘Should content related to caring for transgender and non-binary patients be included in the curriculum?’ as divided by the health professions program

	*n*=	Yes	No	I have no opinion
All	264	161 (61.0%)	40 (15.1%)	63 (23.9%)
Medicine	112	77 (68.8%)	13 (11.6%)	22 (19.6%)
Physiotherapy	33	12 (36.4%)	14 (42.4%)	7 (21.2%)
Midwifery	29	20 (69.0%)	2 (6.9%)	7 (24.1%)
Dentistry	21	11 (52.4%)	4 (19.0%)	6 (28.6%)
Pharmacy	16	11 (68.8%)	0 (0.0%)	5 (31.2%)
Psychology	14	5 (35.8%)	1 (7.1%)	8 (57.1%)
Nursing	14	9 (64.3%)	2 (14.3%)	3 (21.4%)
Paramedics	12	5 (41.7%)	3 (25.0%)	4 (33.3%)
Dietetics	6	6 (100.0%)	0 (0.0%)	0 (0.0%)
Other Healthcare professions*	7	5 (71.4%)	1 (14.3%)	1 (14.3%)

*Other Healthcare professions: Electroradiology (*n* = 2), Speech therapy with audiology (*n* = 1), Public health (*n* = 1), Biomedicine (*n* = 1), Medical analytics (*n* = 1), Occupational therapy (*n* = 1)

**Table 3:** Answers to the question: ‘Should content related to caring for transgender and non-binary patients be included in the curriculum?’ as divided by year of studies

	All	I	II	III	IV	V	VI
*n =*	264	51	50	44	46	48	25
Yes	161 (61.0%)	34 (66.7%)	28 (56.0%)	28 (63.6%)	20 (43.5%)	34 (70.8%)	17 (68.0%)
No	40 (15.2%)	6 (11.8%)	6 (12.0%)	5 (11.4%)	17 (37.0%)	4 (8.3%)	2 (8.0%)
I have no opinion	63 (23.9%)	11 (21.6%)	16 (32.0%)	11 (25.0%)	9 (19.6%)	10 (20.8%)	6 (24.0%)

### Teaching format and time commitment for TGNB healthcare education

**Fig. 1:**
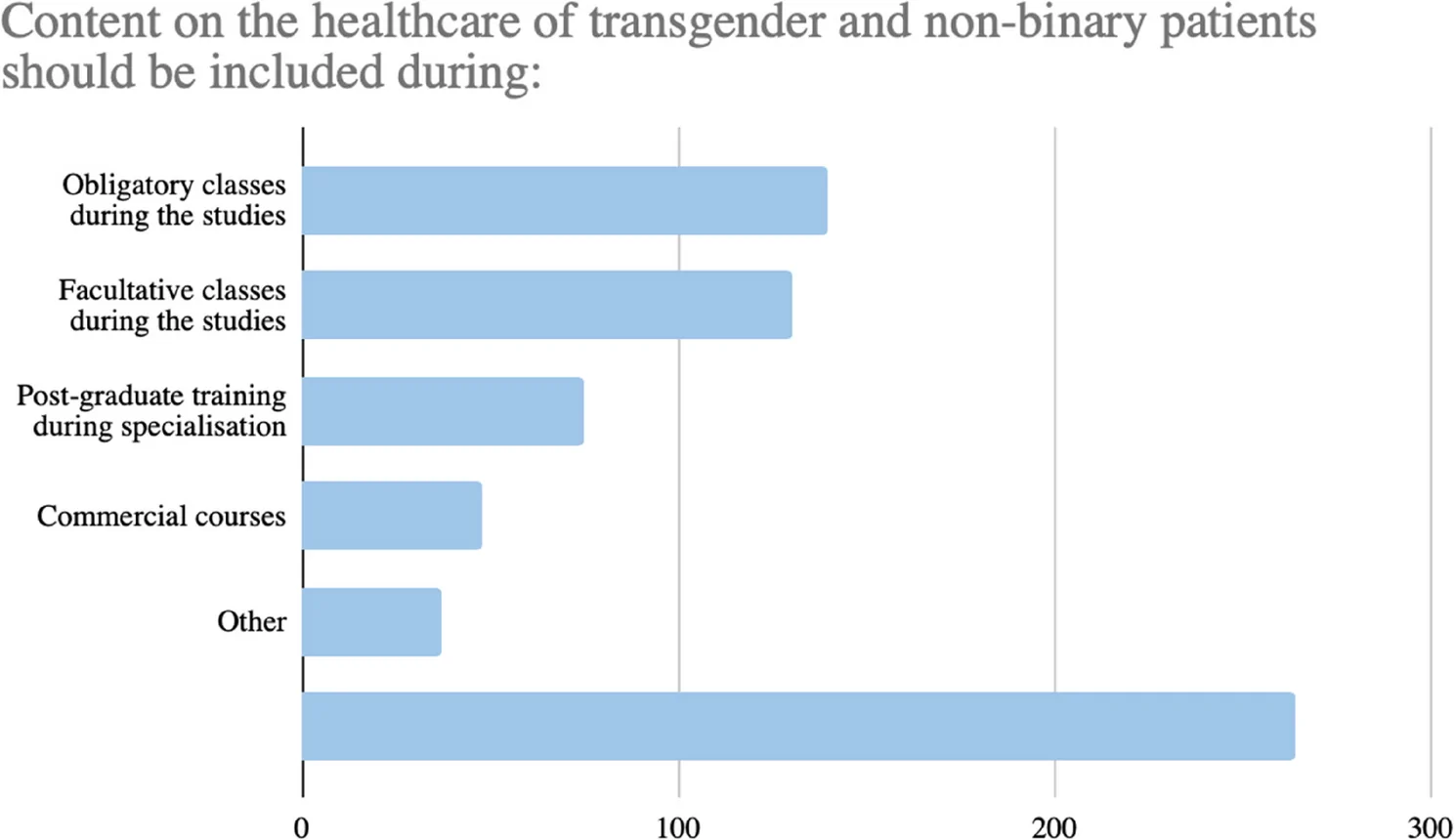
Opinions about the type of training that should include TGNB healthcare education. As “Other”, Some respondents highlighted that such content should never be present

**Fig. 2:**
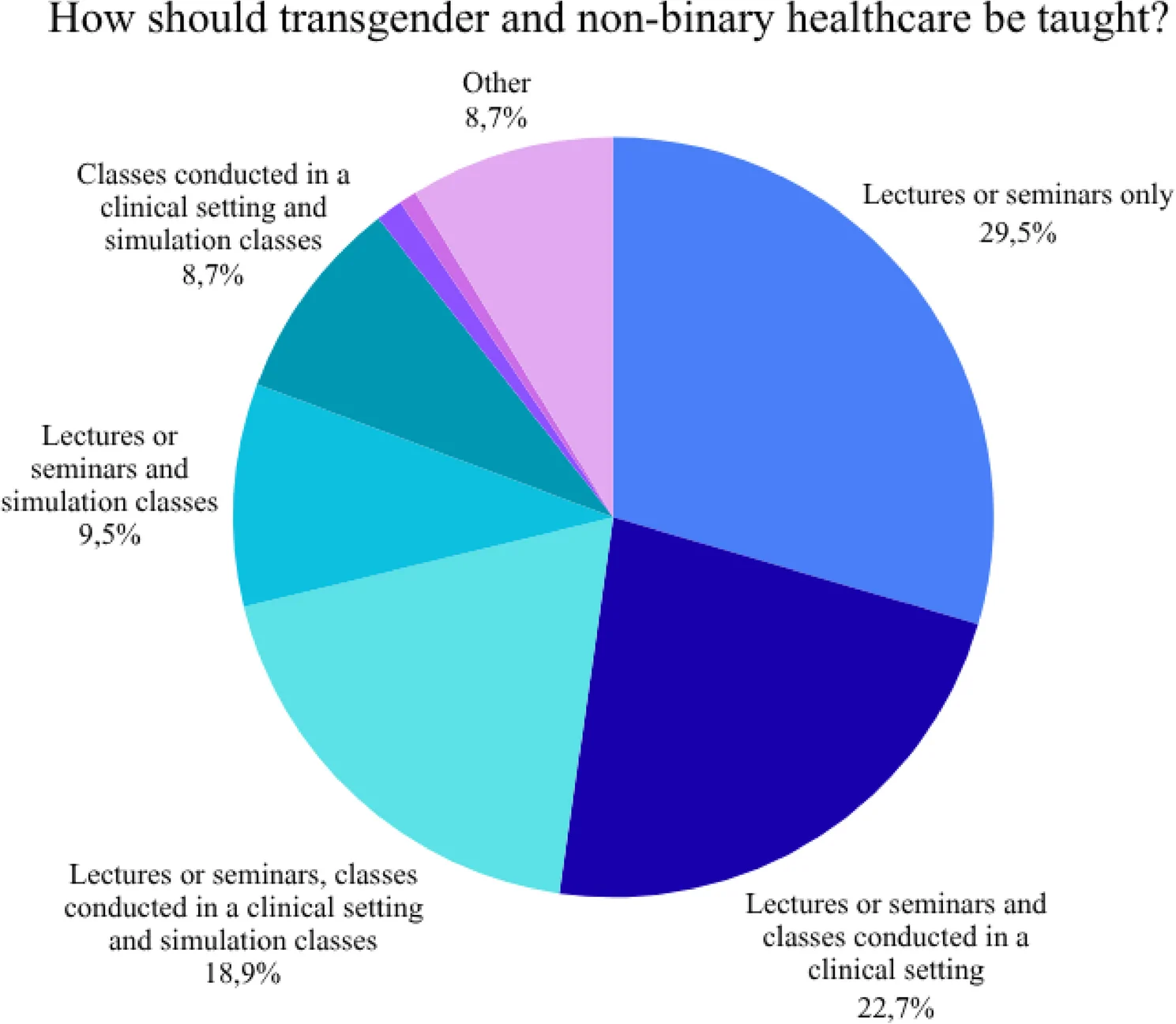
Opinions about the type of lessons that should include TGNB healthcare education

**Fig. 3:**
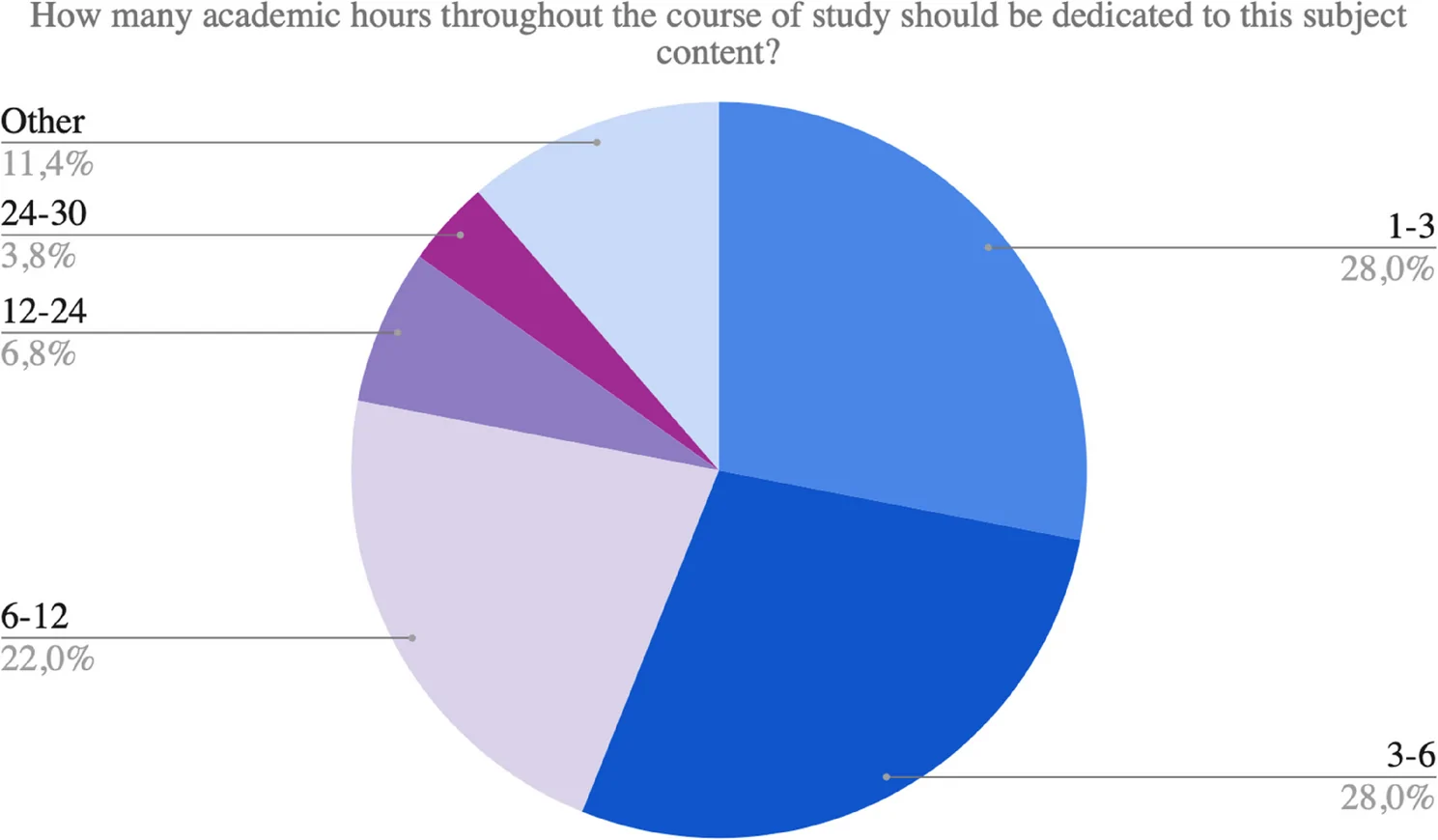
Number of hours devoted for TGNB healthcare education during studies. Among thirty people who selected “Other”, the most common response was zero hours

Survey participants could choose more than one answer to the question regarding the type of classes during which this content should be included. Mandatory courses constitute a compulsory component of the curriculum imposed on all students with a prescribed number of hours, whereas elective courses, while still formally required for the completion of the academic programme, afford students greater autonomy in subject selection. One hundred and forty (53.0%) students answered that TGNB healthcare education should be taught during obligatory classes, whereas 130 (49.2%) thought it would be more appropriate for facultative classes, within the core curriculum. The minority would allocate that content within the postgraduate or commercially available training. The complete data were presented in Fig. 1. The most frequently selected preferred teaching format was lectures or seminars only, chosen by 29.5% of respondents. Sixty people (22.7%) chose the option with additional classes conducted in a clinical setting, and 50 (18.9%) would additionally add simulation classes to the curriculum. Detailed data were presented in Fig. 2. Regarding the amount of time that should be devoted to these topics during the course of study, the most frequently indicated answers were one to three hours (28.0%) and three to six hours (28.0%). The responses of all students were shown in Fig. 3.

**Fig. 4:**
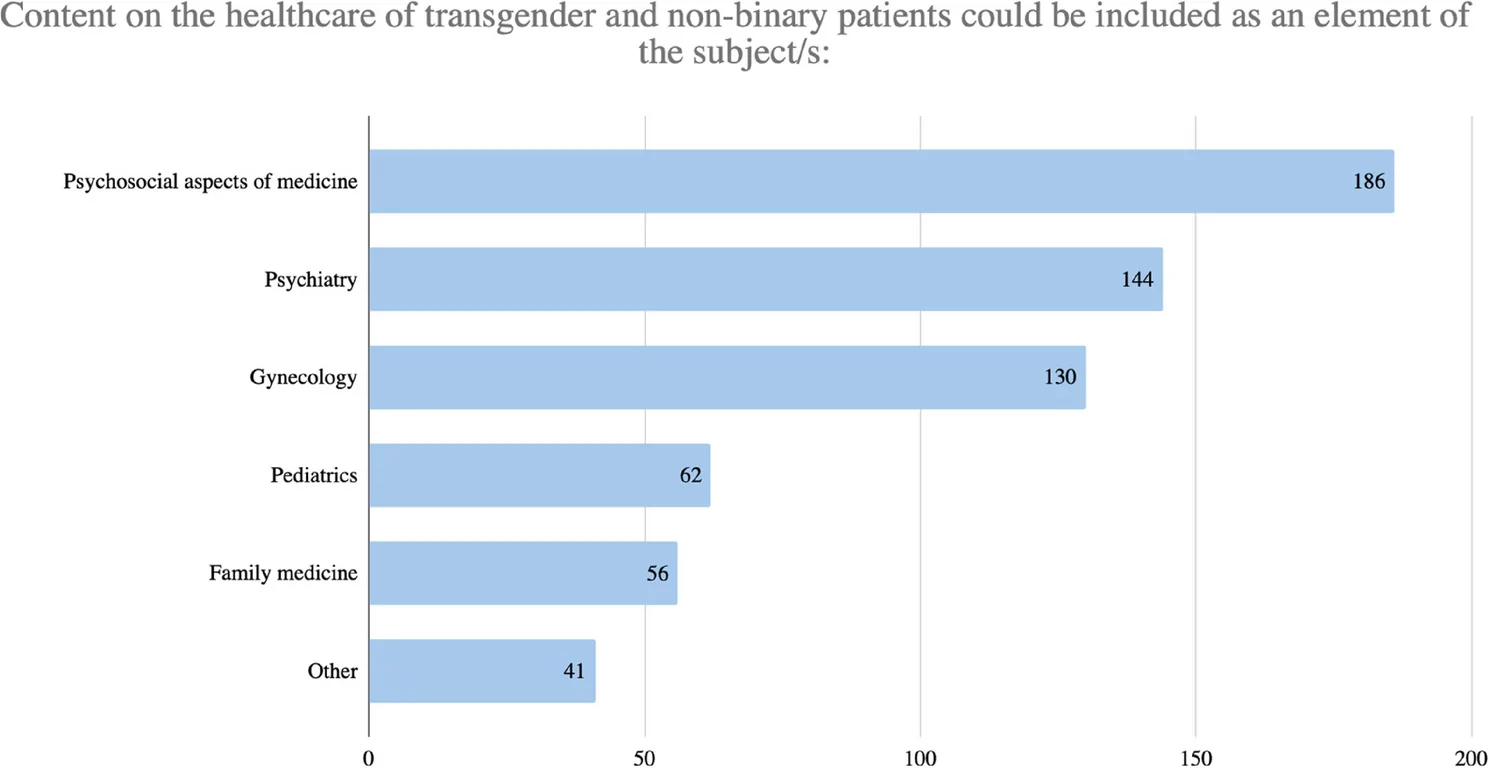
The subject that could include TGNB healthcare content. Within the “Other” option, the most frequent answer was “none”

One hundred and ninety-six (74.2%) respondents believed that content related to healthcare for TGNB people should be taught as a part of existing subject/s, e.g., gynecology. Teaching it as a separate subject was supported by forty-two participants (15.9%). The subjects most frequently identified as suitable for teaching TGNB healthcare education were psychosocial aspects of medicine- one hundred and eighty-six respondents (70.5%), psychiatry- one hundred and forty-four respondents (54.5%), and gynecology- one hundred and thirty respondents (49.2%). The responses were presented in Fig. 4.

### The most relevant topics and self-assessment of knowledge about healthcare for TGNB patients

**Fig. 5:**
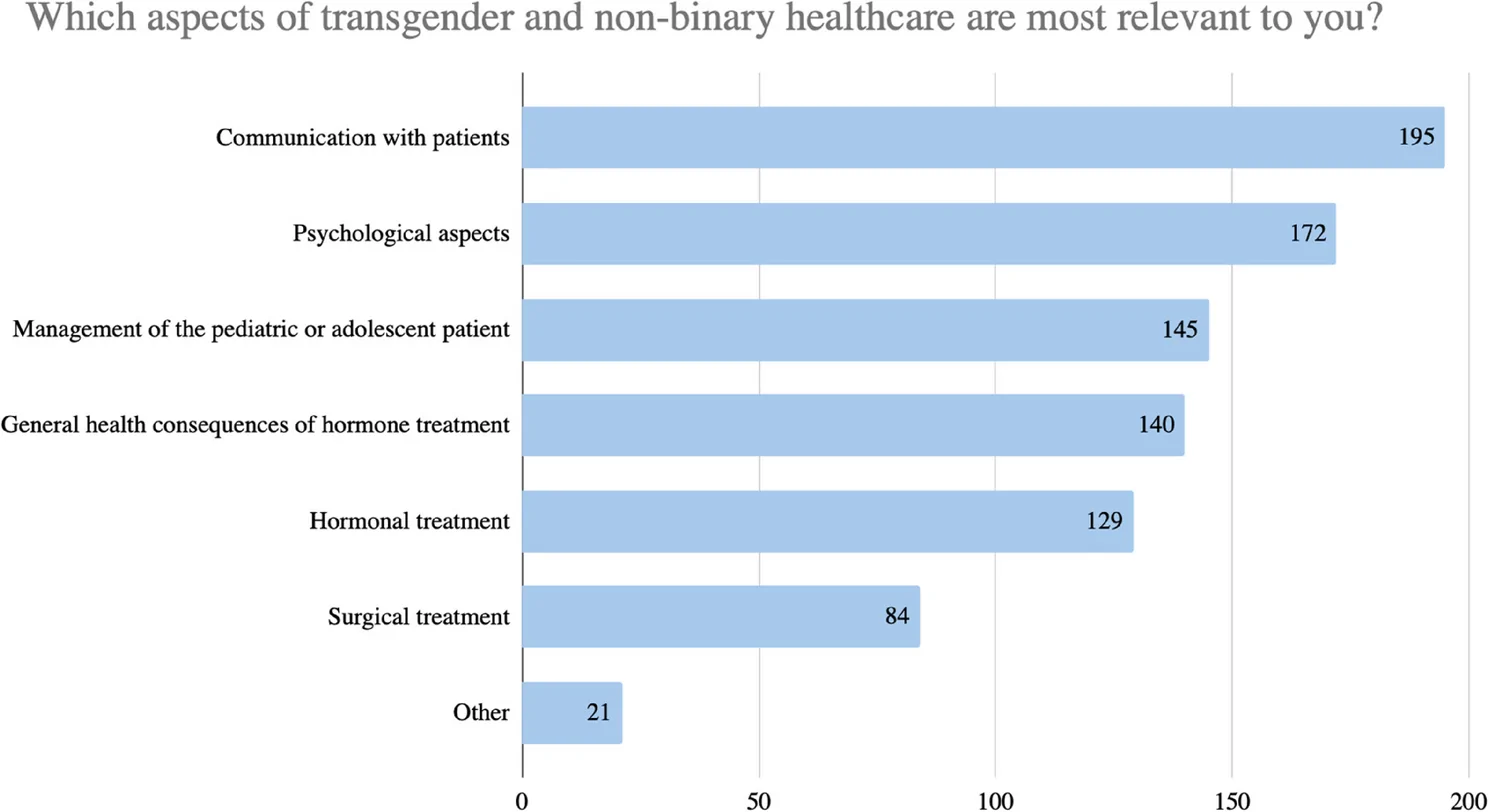
Most relevant aspects of TGNB healthcare education

Participants could select multiple answers to the question about which content was most relevant to them. The most common responses were communication with patients (73.9%), psychological aspects (psychosocial dimensions of care for transgender people, including gender dysphoria, the transition process, and basic psychotherapeutic communication skills with TGNB patients) (65.2%), management of the pediatric or adolescent patient (54.9%) and general health consequences of hormone treatment (53.0%). The data were presented in Fig. 5.

**Fig. 6:**
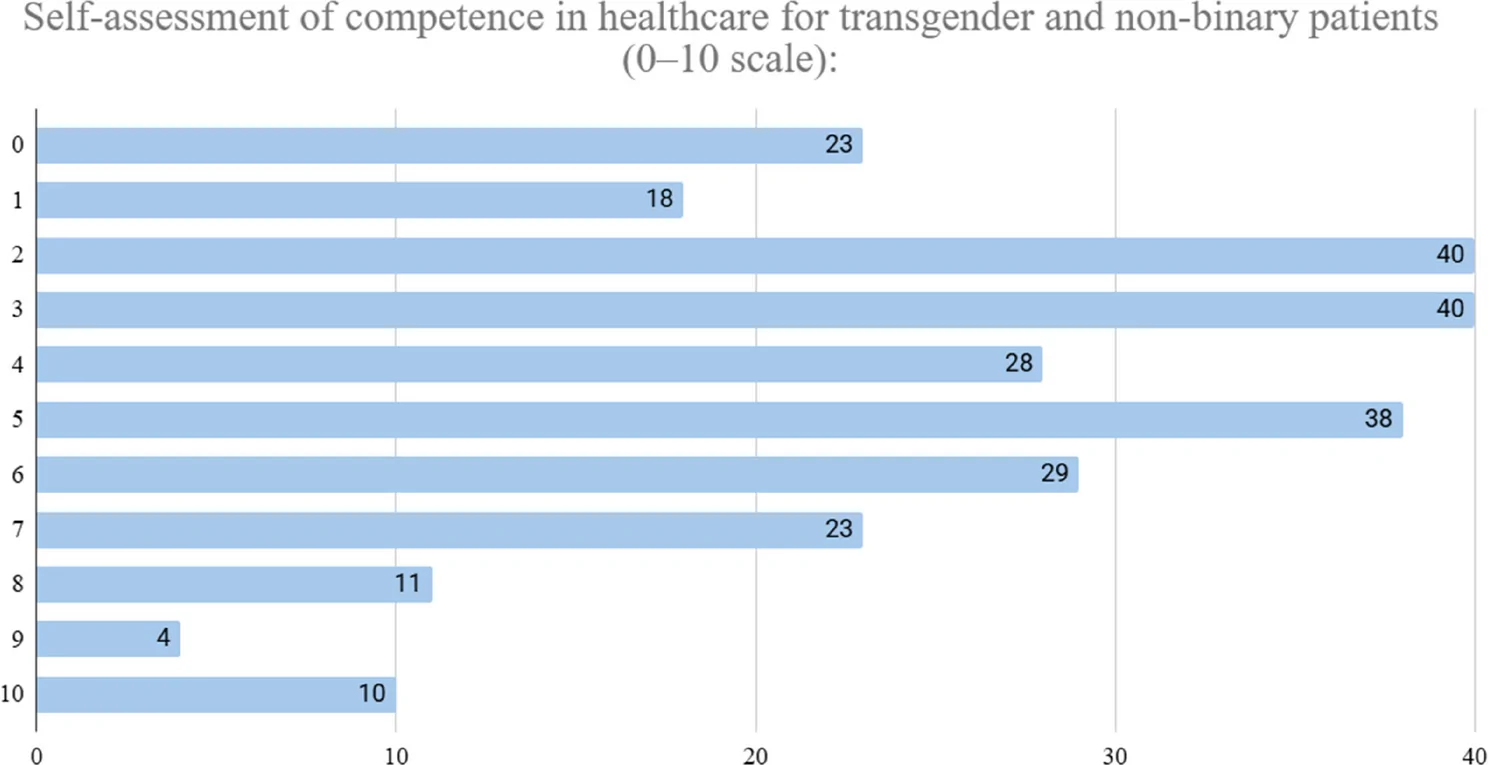
Self-assessment of competence in healthcare for TGNB

Self-rated competency in providing healthcare for TGNB patients, assessed on a 0–10 scale (0 = no competency, 10 = very high competency), had a median score of 4 and an average score of 4.09. The self-assessed competence scores were distributed as follows: 0–8.7%, 1–6.8%, 2–15.2%, 3–15.2%, 4–10.6%, 5–14.4%, 6–11.0%, 7–8.7%, 8–4.2%, 9–1.5%, 10–3.7%. The breakdown of self-assessed knowledge on TGNB healthcare according to year of study revealed the following means: I year of study- 4.33, II year of study- 4.16, III year of study- 3.59, IV year of study- 4.37, V year of study- 4.23 and VI year of study- 3.52. The breakdown by field of study showed the following means: medicine- 3.91, physiotherapy- 4.55, midwifery- 4.03, dentistry- 3.76, pharmacy- 3.75, psychology- 4.0, nursing- 5.14, paramedics- 4.67, dietetics- 2.33, other healthcare professions- 5.29. The results were presented in Fig. 6.

**Fig. 7:**
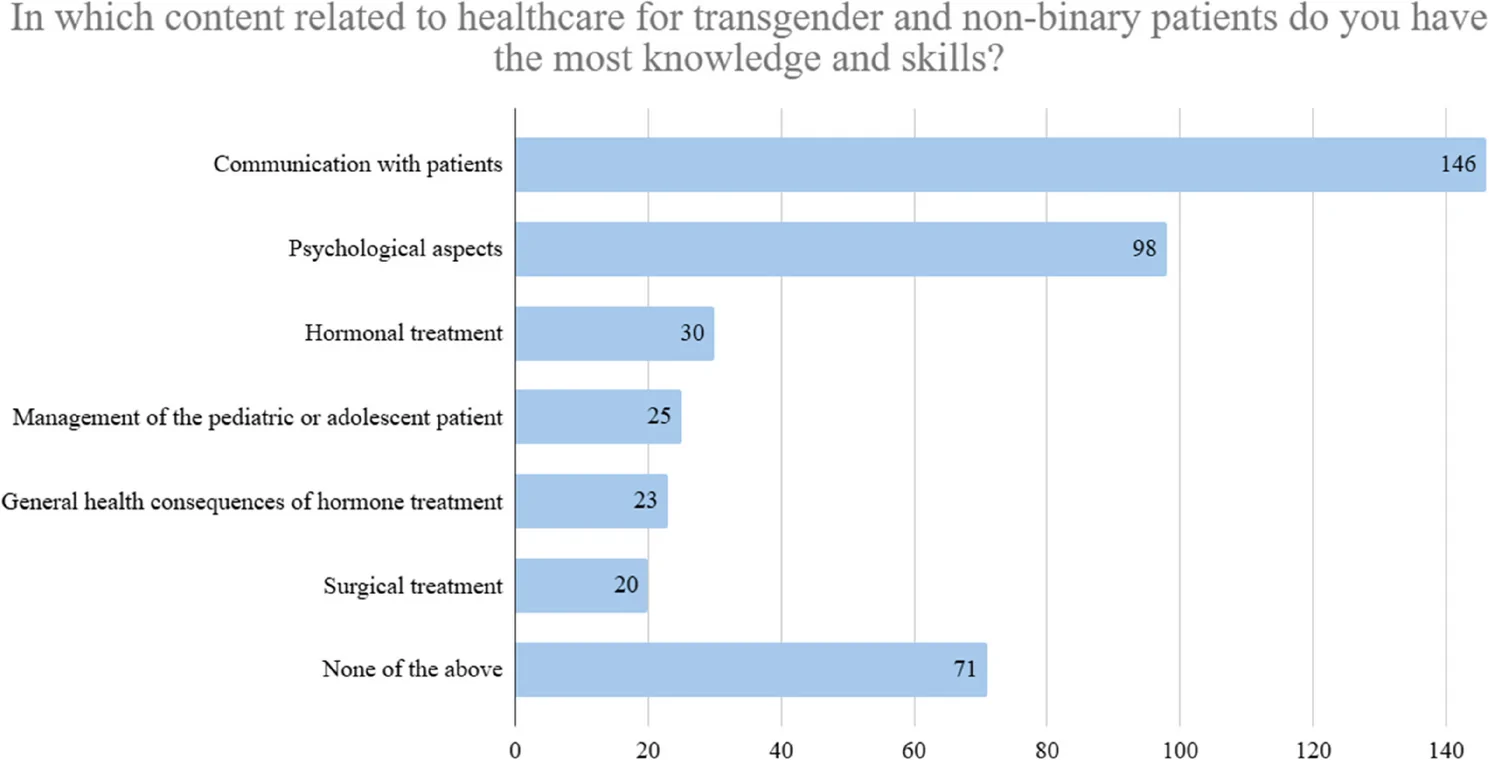
TGNB healthcare education areas in which students reported the highest level of self-assessed knowledge and skills

**Fig. 8:**
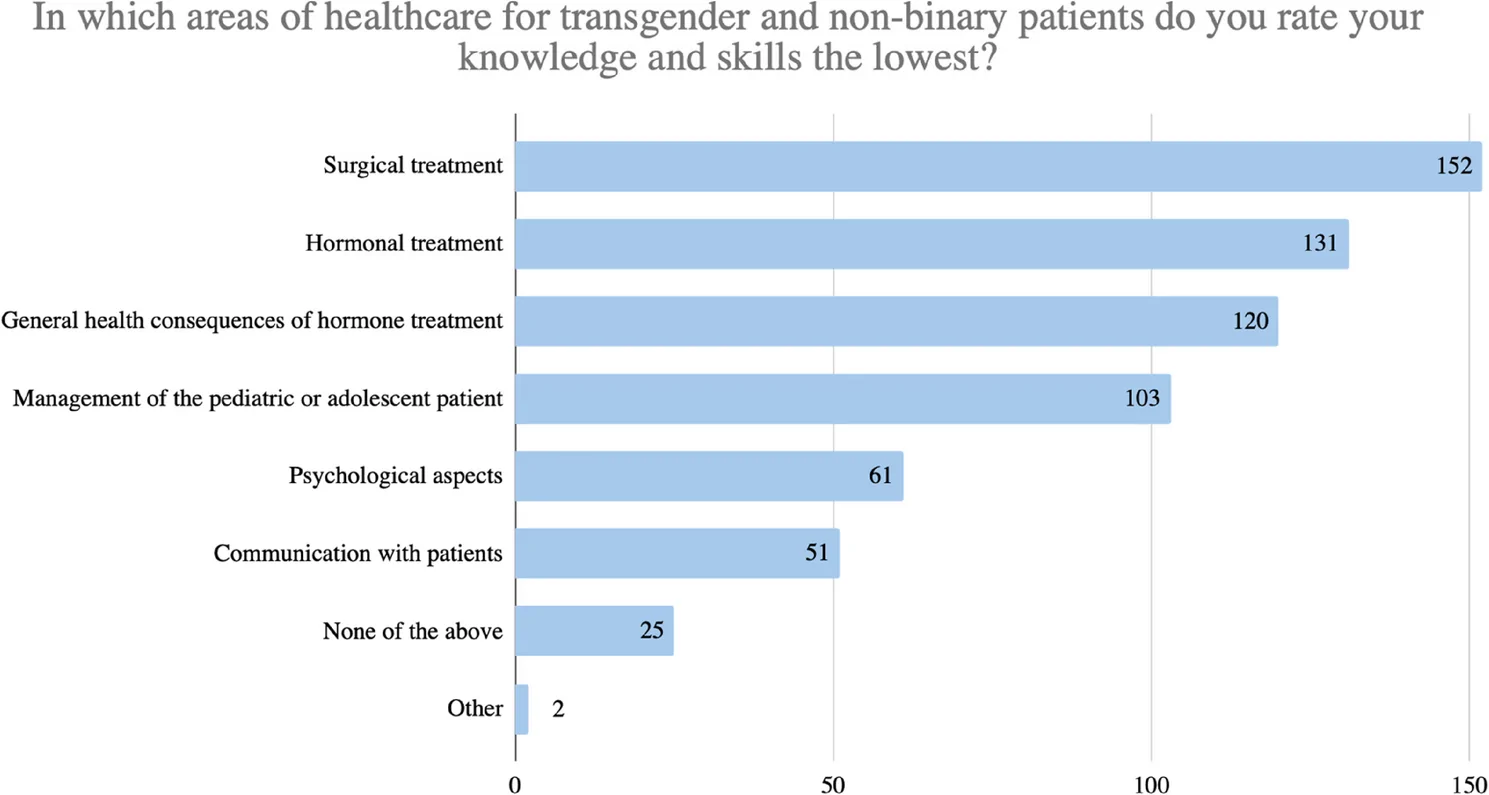
TGNB healthcare education areas in which students reported the lowest level of self-assessed knowledge and skills

The highest levels of self-assessed competence were reported in communication with patients (55.3%) and psychological aspects (37.1%), however over a quarter of participants (26.9%) indicated “none of the above”. Respondents could select multiple answers. The data were shown in Fig. 7. In contrast, participants considered their competency to be lowest in the area of surgical management (59.1%), hormonal treatment (50.0%), general health consequences of hormonal treatment (e.g., complications, primary care) (46.2%), and the management of pediatric or adolescent patients (39.8%). The answer “none of the above” was indicated by less than one in ten respondents (9.5%). The results were presented in Fig. 8.

### Sources of knowledge and previous experience with the healthcare for TGNB patients

**Fig. 9:**
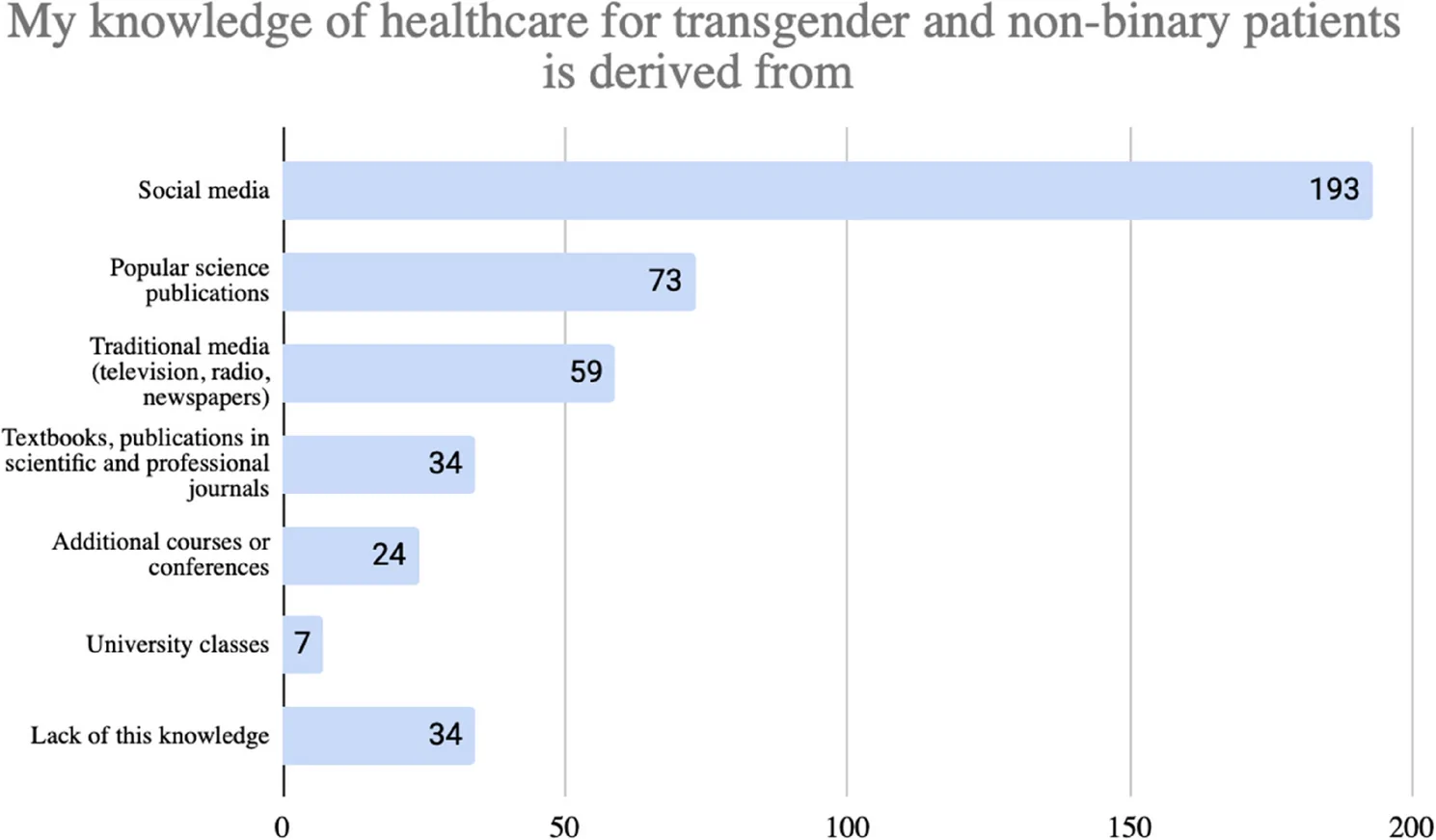
Sources of knowledge on TGNB healthcare

The respondents most frequently indicated that their knowledge regarding healthcare for TGNB patients was derived from social media (73.1%). Other commonly reported sources included popular science publications (27.7%) and traditional media such as television, radio, and newspapers (22.3%). Less frequently, respondents reported obtaining such knowledge from textbooks or scientific journal publications (12.9%) and additional courses or conferences (9.1%). 2.7% of participants reported that their knowledge was derived from courses they took during their academic career. Notably, 12.9% of respondents reported having no knowledge regarding healthcare for TGNB patients. The results were presented in Fig. 9. Only 12.1% of participants confirmed that they had been exposed to TGNB healthcare education, whereas the overwhelming majority of respondents (87.9%) said that they had not. Similarly, most respondents reported no direct contact with TGNB patients during their studies (86.0%). Only 14.0% of participants indicated that they had previous experience with such patients in the course of their academic training.

### Perception of TGNB healthcare issues in relation to future work in the healthcare system

In response to the questions regarding preparedness for interactions with TGNB patients in their prospective professional practice, 19.7% of respondents expressed a sense of preparedness, while 45.5% indicated that they did not feel prepared. A further 34.8% of the respondents stated that they were unsure about their level of preparedness. With regard to attitudes toward medical documentation practices, over half of respondents (55.7%) agreed that medical documentation should include information about a patient’s preferred pronouns or gender identity. In contrast, 24.6% of participants disagreed with this statement, while 19.7% of respondents expressed uncertainty. Regarding prior exposure to TGNB healthcare education, 53.0% of respondents reported prior engagement with these topics. A total of 31.4% of respondents stated that they had not previously considered TGNB content but were beginning to identify new educational needs in this area. Conversely, 15.2% of participants reported that they had not previously considered this topic and they believed this subject to be unnecessary.

## Discussion

The study’s aim was to examine attitudes and self-assessed knowledge levels of healthcare students regarding TGNB healthcare education. This approach enabled the identification of specific educational needs in this area. Our findings show that students hold a positive attitude toward TGNB-related healthcare education. At the same time, the majority of respondents have no prior curricular education on that topic. Instead, social media have become a key source of information on TGNB topics. That implies that current curricula should be expanded by incorporating both theoretical and practical knowledge regarding TGNB. Further research is needed to evaluate the quality of TGNB-related information shared on social media. The findings could serve as a valuable resource for developing new curricula, not only in Poland but also in other European countries.

The lack of knowledge among medical personnel about the care of TGNB individuals, resulting from a lack of content on this topic in medical and health-related curricula, leads to inequality in medical care for these patients [19–21]. To our knowledge, only two studies have previously addressed this topic among students in Poland [22, 23]. Moreover, examining these perspectives in a traditionally Catholic-based society—where systemic barriers to sex education often persist—allows for a deeper understanding of how future physicians develop their clinical attitudes toward TGNB patients. Van Heesewijk et al., in a systematic review of 46 studies on TGNB healthcare content in medical education (2009–2021), noted that ‘most of the current research reports are from the US and Canada’, with no studies originating from Eastern European countries [24].

The proportion of respondents identifying as non-binary (3.4%) and transgender (1.5%) was notably higher than population-level prevalence estimates. This can be related to greater awareness of gender diversity in an academic environment and greater willingness of TGNB to participate in health-related research [25]. Although this finding is a secondary focus of the study, it underlines the importance of considering sample characteristics when interpreting attitudes towards TGNB healthcare education.

Our findings indicate that social media currently represent the main source of information regarding TGNB healthcare education. At the same time, medical students have a feeling of a lack of appropriate and reliable sources of information related to LGBTQ healthcare [26]. From 73.1% (our study) up to even 89.6% (Martins et al.) respondents reported the source of their knowledge as social media [27]. There is a huge disparity between social media and other sources as the next popular source of knowledge was chosen by 27.7% of those surveyed. Further research is required to assess the quality of TGNB-related information shared on social media. In our study, scientific publications represented a valuable source of information for the Polish community, which supports the importance of creating scientific materials.

Previously published studies found that medical students whose curricula lacked TGNB health education supported its inclusion in the teaching program [27–29]. Our cohort of students noted a similar rate of TGNB healthcare education inclusion compared with other undergraduate education programs (54.6%- 61.0%) [27, 30]. Recognition of the importance of TGNB health education has been reported to be as high as 95% in a Canadian study [28, 29].

For respondents who indicated interest in TGNB curricular inclusion, lectures and seminars were chosen most often, while others preferred simulation. This is consistent with existing literature on attitudes regarding TGNB healthcare education [31–33]. The predominant preference for lectures and seminars among our cohort likely comes from a long-standing exposure to traditional didactic models in the national educational system [34]. This preference may reflect limited exposure to interactive, competency-based teaching strategies, such as OSCEs, which were only introduced at Polish medical universities within the last decade [35].

Currently, lessons on LGBTQ healthcare take up very little time in medical school programs and the length of the classes varies depending on the format [36]. The time seems very subjective as the majority of students (81.0%) want to continue learning about this topic after education intervention [37]. In the context of countries where TGNB healthcare education is absent from the medical curriculum, the most important aspect appears to be the inclusion of any TGNB healthcare content rather than the amount of time devoted to it. This conclusion is also supported by the findings of Ellaway et al. [38]. However, in countries where TGNB healthcare education is already incorporated, the discussion has already shifted toward the extent of curricular hours. A 2021 survey of U.S. and Canadian medical schools found that an average of 11 h was devoted to LGBT-related content across the curriculum. This amount was still falling short of the number of hours based on recommended LGBTQI + health competencies from the Association of American Medical Colleges [39].

The literature reports that teaching transgender health and related topics was embedded mainly within clinical subjects such as family medicine, psychiatry, internal medicine, endocrinology, urology, emergency medicine, surgery, plastic surgery, otolaryngology, and pediatrics [12, 31, 40, 41]. In contrast, the participants of our study decided that transgender health issues would be best fitted within the course concerning psychosocial aspects of medicine. This may reflect a lack of knowledge regarding the broader scope of TGNB healthcare. A more effective approach to teaching TGNB healthcare may be to combine theoretical instruction with its integration into practical clinical training. It has been suggested that medical students should be taught both general and gynecological issues related to TGNB patients [37]. The hierarchy of issues identified by our cohort as priorities for learning about TGNB healthcare (communication > clinical aspects) may not reflect actual educational needs, but rather recognition of universally valued clinical skill. Communication can be an easy factor to identify as important regardless of prior exposure to TGNB-specific content. Given that fewer than 3% of respondents reported having received formal education, these rankings should be interpreted as a reflection of respondents’ existing frame of reference rather than a reliable indication of actual educational priorities. In a study by Martins et al., in which 249 Pakistan medical students participated, the most frequently selected issues in the care of TGNB were counseling (90.0%), clinical examinations (87.2%), and medical history (85.2%) [27]. The TGNB healthcare education is often associated with LGBTQ healthcare education. According to a 2011 study in American medical schools, the most frequently taught LGBT topics included sexual orientation, HIV, gender identity, and sexually transmitted infections (STIs), which can also be important issues to discuss in TGNB context [16].

The findings regarding contact with transgender patients vary widely. Findings from the U.S. studies indicated that 60.0% to 80.0% of students had worked with at least one transgender patient [21, 25]. Nevertheless, some studies reported that despite simulation training, 68.0% of students considered themselves incompetent in that area [33]. The huge difference between U.S and Polish medical education systems is seen here, as 86% of students reported no direct contact with TGNB patients during their studies and 45.5% indicated that they did not feel sufficiently prepared to care for TGNB individuals.

Another important issue that has been observed in medical clinics has been correct use of pronouns. In our survey, more than half of the respondents (55.7%) agreed that information about a patient’s preferred pronouns or gender identity should be included in the medical documentation. An important question that remains to be addressed is whether this competency is primarily required for doctors or should be incorporated more broadly across other healthcare professions. A study performed among nursing students found that the correct use of pronouns while conducting a nursing assessment, and deciding which hospital room would be most appropriate for the TGNB patient were problematic [30]. Findings from the studies, indicated however, that medical students uniformly agreed that questions about gender identity and the use of appropriate pronouns were important [12, 19, 42].

The relatively high levels of self-confidence, perceived knowledge, and willingness to work with TGNB patients observed among respondents may be attributable to several factors. The increasing availability of TGNB content across digital platforms may have facilitated independent exploration of the topic outside of formal academic settings [43]. Students may feel confident in their knowledge while relying primarily on informal sources, which do not always align with current clinical guidelines and evidence-based medical practice. Furthermore, the heightened sociopolitical discourse surrounding TGNB issues in Poland in recent years such as anti-LGBTQ campaigns may have prompted an increasing number of individuals to actively seek information independently and form their own opinions on the subject [44].

It has been shown that teaching health-related content for transgender people improves knowledge and increases awareness and clinical confidence among medical students, thereby increasing comfort in their relationships with patients [20, 37, 39, 40]. In the long term, it can also increase knowledge about gender dysphoria, as well as expertise in hormone therapy, gender-affirming surgery, and mental health [21]. Increased training time and contact with transgender patients correlated with greater comfort in caring for them [16]. The inclusion of gender identity and sexuality issues in medical school curricula is also supported by the improvement of LGBTQ patients’ experience with and better access to healthcare [37].

Strengths and limitations.

The study addressed a gap in existing scientific literature regarding medical education of the topic of TGNB in eastern Europe countries. Our research has important limitations to note. The research did not quantitatively assess the specific extent of knowledge previously acquired regarding TGNB. The study relied only on self-perceptions of knowledge that may be different from objective clinical assessments. The absence of longitudinal data prevents an assessment of the long-term efficacy of educational interventions.

## Conclusions

Implementing TGNB healthcare education is a direction supported by medical faculties students. Developing any type of TGNB healthcare education is essential to ensure that future medical practitioners can provide affirming and appropriate care to TGNB patients. Curricular interventions addressing TGNB healthcare should extend beyond communication and psychosocial competencies to include valuable clinical skills, such as the appropriate selection and management of hormone therapy. Further research is needed to evaluate the quality of TGNB-related information shared on social media.

## Supplementary Information

Additional File 1: Survey on knowledge and educational needs regarding healthcare for transgender and non-binary patients.

Additional File 2: Pairwise comparisons of student responses across years of study.

## Data Availability

Data generated and analysed during the current study are available from the corresponding author on reasonable request.

## Abbreviations

TGNB: Transgender and non-binary
NBGQ: Non-binary and genderqueer
LGBT: lesbian, gay, bisexual, and transgender
LGB: lesbian, gay, and bisexual
LGBTQ: lesbian, gay, bisexual, transgender and queer/questioning
WHO: The World Health Organization
AAMC: the Association of American Medical Colleges
STI: sexually transmitted infection
HIV: human immunodeficiency virus
