# Supplementary material for: Exploring the educational needs of transgender and non-binary healthcare among Polish medical students
Source: BMC Med Educ. 2026 Aug 14;26:1827. doi: 10.1186/s12909-026-10139-4 (PMC13749859; doi:10.1186/s12909-026-10139-4)
Supplement: Supplementary file 1 [file 12909_2026_10139_MOESM1_ESM.pdf]

Educational Needs in Healthcare – Transgender and Non-Binary Patients

Survey on Knowledge and Educational Needs Regarding Healthcare for Transgender and Non-Binary Patients

Good morning!
We are a team from the Department of Peadiatric and Adolescent Gynecology at the Medical University of Lublin. The purpose of this survey is to gather information regarding the knowledge and educational needs of medical students in the field of healthcare for transgender and non-binary patients. The insights gained will help identify potential educational gaps in this area and create new training opportunities.
Please answer the questions below. Completing the survey will take approximately 7 minutes. The survey is anonymous, and participation is completely voluntary. To ensure the survey is valid, an answer is required for each question. The research team guarantees that the nature of the responses will not allow any data to be linked to the participants' identities. The results will be compiled and presented collectively. The information obtained will help better adapt the curriculum to your needs.
Contact Information:
If you have any concerns regarding this study, you may contact a representative of the Bioethics Committee of the Medical University of Lublin or a representative of the research team: Joanna Brodowska.

Please note that for the purpose of this survey, the word "patient" (and its grammatical context in the Polish version) is intended to be gender-neutral.

1. Field of study:

a) Medicine
b) Dentistry
c) Health sciences – Nursing
d) Health sciences – Midwifery
e) Health sciences – Emergency Medical Services / Paramedicine
f) Health sciences – Physiotherapy
g) Health sciences – Dietetics
h) Pharmacy
i) Other: …………………………………

2. Year of study (for Health Sciences students, please indicate the year chronologically from the beginning of studies – without dividing into bachelor's and master's studies):

a) 1

b) 2

c) 3

d) 4

e) 5

f) 6

3. The gender you identify with is:

a) Consistent with the sex assigned at birth (biological sex)

b) Inconsistent with the sex assigned at birth (biological sex) – transgender

c) Non-binary

d) Other options (please specify) …………

e) Prefer not to answer

4. Should content related to caring for transgender and non-binary patients be included in the curriculum?

a) Yes

5. Why? …………

b) No

6. Why? …………

c) No opinion

7. I believe that content regarding care for transgender and non-binary patients should be included in:

a) Obligatory classes during studies

b) Facultative classes during studies

c) Postgraduate training during specialisation

d) Commercial courses for interested individuals

e) Other option/comment: …………………………………

8. How many academic hours throughout the course of study should be dedicated to this subject content?

a) 1-3

b) 3-6

c) 6-12

d) 12-24

e) 24-30

f) Other number:……………..

9. In what form should this content be taught during studies?

a) As a separate subject

b) As part of an existing subject/subjects, e.g. gynecology, etc.

10. Within which subjects could content regarding care for transgender and non-binary patients be included?

a) As part of gynecology

b) As part of psychiatry

c) As part of psychosocial aspects of medicine

d) As part of family medicine

e) As part of pediatrics

f) Other: …………………………

11. How should transgender and non-binary healthcare be taught?

a) Lectures and seminars only

b) Simulation classes only

c) Classes conducted in a clinical setting only

d) Lectures or seminars and simulation classes

e) Classes conducted in a clinical setting and simulation classes

f) Lectures or seminars and classes conducted in a clinical setting

g) Lectures or seminars, classes conducted in a clinical setting and simulation classes

h) Other option/comment:…………

12. Which aspects of transgender and non-binary healthcare are most relevant to you?

a) Communication with patients

b) Hormonal treatment

c) Surgical treatment

d) General health consequences of hormone treatment

e) Psychological aspects

f) Management of the pediatric or adolescent patient

g) Other option/comment: ……………………………………….

13. On a scale from 0 to 10 (where 0 means no knowledge and 10 means a very strong sense of knowledge), how would you rate your knowledge and skills regarding healthcare for transgender and non-binary patients?

a) 0

b) 1

c) 2

d) 3

e) 4

f) 5

g) 6

h) 7

i) 8

j) 9

k) 10

14. In which content related to healthcare for transgender and non-binary patients do you have the most knowledge and skills?

a) Communication with patients

b) Hormonal treatment

c) Surgical treatment

d) General health consequences of hormone treatment

e) Psychological aspects

f) Management of the pediatric or adolescent patient

g) None of the above

h) Other option/comment: ………………………………………….

15. In which areas of healthcare for transgender and non-binary patients do you rate your knowledge and skills the lowest?

a) Communication with patients

b) Hormonal treatment

c) Surgical treatment

d) General health consequences of hormone treatment

e) Psychological aspects

f) Management of the pediatric or adolescent patient

g) None of the above

h) Other option/comment: ……………………………………….

16. My knowledge of healthcare for transgender and non-binary patients is derived from:

a) University classes

b) Traditional media (television, radio, newspapers)

c) Social media

d) Popular science publications

e) Textbooks, publications in scientific and professional journals

f) Additional courses or conferences

g) Lack of this knowledge

17. During your studies so far, have you been taught content regarding healthcare for transgender and non-binary patients?

a) Yes
b) No

18. During your studies so far, have you had contact with transgender or non-binary patients?

a) Yes
b) No

19. As a future healthcare professional, do you feel prepared for contact with transgender and non-binary patients?

a) Yes
b) No
c) I am not sure

20. Do you think that medical documentation should include information about preferred pronouns or the gender with which the patient identifies?

a) Yes
b) No
c) I am not sure

21. Have you ever considered the healthcare issues for transgender and non-binary patients before?

a) Yes
b) Yes, only sporadically

c) No, but now I am beginning to understand the new educational needs in this area
d) No, I think this subject is unnecessary in medical educations
e) Other option/comment: ……………………………………….

Thank you for completing the survey!
