# Supplementary material for: Exploring the educational needs of transgender and non-binary healthcare among Polish medical students
Source: BMC Med Educ. 2026 Aug 14;26:1827. doi: 10.1186/s12909-026-10139-4 (PMC13749859; doi:10.1186/s12909-026-10139-4)
Supplement: Supplementary file 2 [file 12909_2026_10139_MOESM2_ESM.pdf]

**Comparison**	**p value**	**Significant after Bonferroni correction**
I vs II	0.473	No
I vs III	0.925	No
**I vs IV**	**0.012**	No
I vs V	0.837	No
I vs VI	0.872	No
II vs III	0.727	No
**II vs IV**	**0.015**	No
II vs V	0.313	No
II vs VI	0.602	No
**III vs IV**	**0.018**	No
III vs V	0.753	No
III vs VI	0.891	No
**IV vs V**	**0.003**	**Yes**
**IV vs VI**	**0.029**	No
V vs VI	0.953	No

Overall comparison among all study years: $\chi^2 = 23.75$, df = 10, $p = 0.008$.
